# Radiolabeled Compounds for Diagnosis and Treatment of Cancer

**DOI:** 10.3390/molecules26206227

**Published:** 2021-10-15

**Authors:** Krishan Kumar

**Affiliations:** Laboratory for Translational Research in Imaging Pharmaceuticals, The Wright Center of Innovation in Biomedical Imaging, Department of Radiology, The Ohio State University, Columbus, OH 43212, USA; krishan.kumar@osumc.edu

Nuclear medicine was recognized as a potential medical field a long time ago when ^131^I was used in thyroid cancer patients [1,2,3]. Diagnostics and Therapeutics are the two branches of Nuclear Medicine. Single-Photon Emission Computed Tomography (SPECT) and Positron Emission Tomography (PET) are in vivo molecular imaging modalities which are widely used in nuclear medicine for the diagnosis and follow-up of many major diseases after treatment [4,5]. Combining PET with Computed X-Ray Tomography (CT), PET-CT, enables better diagnosis than with a traditional gamma camera alone. It is a powerful tool that provides unique information on a wide variety of diseases.

These methods use radiolabeled target-specific molecules and biomolecules, including peptides, proteins, protein fragments, and monoclonal antibodies (mAbs) as probes or imaging pharmaceuticals or radiopharmaceuticals. Molecules and biomolecules are labeled with metallic or non-metallic radionuclides with the desired emission type and half-lives for the intended application. Imaging pharmaceuticals are being used routinely in cardiology, neurology, and oncology, etc. Their design and development is a rather interdisciplinary process covering many different areas of science: chemistry, radiochemistry, pharmaceutical, analytical, medicine, engineering, regulatory, etc.

The use of radionuclides for therapeutic applications was reported some time ago [1,2,3]. Several radionuclides have been used successfully for the treatment of many benign and malignant disorders [6]. For example, several new radionuclides and radiopharmaceuticals have been developed for the treatment of metastatic bone pain and neuroendocrine and other malignant or non-malignant tumors. Radioimmunotherapy is a targeting therapy for cancer that uses monoclonal antibodies (mAbs) labeled with a radionuclide directed against tumor-associated antigens. The ability for the antibody to specifically bind to a tumor-associated antigen increases the dose delivered to the cancer cells specifically, while decreasing the dose to normal tissues.

The concept of Theranostics has an integrated approach to diagnosis and therapy. A targeting vector is radiolabeled with a therapeutic radionuclide which also emits radiation for imaging. Alternatively, the targeting vector is labeled either with a diagnostic or a therapeutic radionuclide with similar chemical properties. One of the classic examples of theranostics is the use of ^68^Ga-labeled tracers for diagnosis followed by therapy using a therapeutic radionuclide, i.e., ^177^Lu, etc. In addition to their diagnostic and therapeutic applications in nuclear medicine, radiolabeled compounds are powerful tools for in vitro/in vivo evaluation during discovery and preclinical development and to evaluate the in vivo pharmacokinetics and pharmacodynamics of potential drug candidates.

Numerous radiopharmaceuticals based on,^11^C, ^64^Cu, ^18^F, ^67^Ga, ^68^Ga, ^111^In, ^123^I, ^125^I, ^131^I, ^177^Lu, ^13^N, ^223^Ra, ^153^Sm, ^99m^Tc, ^201^Tl, ^133^Xe, and ^90^Y, radionuclides have been approved by the Food and Drug Administration (FDA) for various diagnostics and therapeutics applications [7]. Significant research is ongoing worldwide for use of novel radionuclides and radiolabeled molecules and biomolecules in oncology, neurology, and cardiology for imaging and therapy. A large number of human clinical trials using radionuclides and radiopharmaceuticals have been completed in the past and still are ongoing. Details of these clinical trials can be found in the Clinical Trials database (www.clinicaltrial.gov, accessed on 29 September 2021) published by the US National Library of Medicine of NIH.

The objective of the Special Issue entitled “Radiolabeled Compounds for Diagnosis and Treatment of Cancer” was to focus on all aspects of design, characterization, evaluation, and development of novel radiolabeled compounds for the diagnosis and treatment of cancer and the application of new radiochemistry and methodologies for the development of novel radiolabeled compounds. The Special Issue includes eleven outstanding papers, including seven research and four review articles. The following is an overview of these papers.

The main objective of the first paper by Kumar and Woolum was to develop and test a novel reagent, inorganic monochloramine (NH_2_Cl) for radioiodine labeling of new chemical entities and biomolecules, which is cost-effective, easy to make and handle, and is selective to label amino acids, peptides, and proteins. The data presented in this report demonstrate that the yields of the non-radioactive iodine labeling reactions using monochloramine are >70% for an amino acid and a cyclic peptide. The reagent selectively iodinates the tyrosine residue in the biomolecules.

A new squaramide-containing AAZTA^5^ (1,4-bis-(carboxymethyl)-6-[bis-(carboxymethyl)-amino-6-pentanoic-acid]-perhydro-1,4-diazepine) chelator for targeting FAP (Fibroblast Activation Protein) was evaluated by Rosch and coworkers. The ^68^Ga-, ^44^Sc-, and ^177^Lu- AAZTA^5^.SA.FAPi chelates were investigated for their in vitro properties and compared with those of DOTA.SA.FAPi. AAZTA^5^.SA.FAPi and its derivatives showed sub-nanomolar IC_50_ values for FAP and sufficiently high stability in different media.

Vorobyeva et al. evaluated an ankyrin repeat protein (DARPin) Ec1, for imaging of EpCAM (Epithelial Cell Adhesion Molecule) in Triple-Negative Breast Cancer (TNBC). ^125^I and ^99m^Tc-labeled DARPin Ec1 imaging probes retained high binding specificity and affinity to EpCAM-expressing MDA-MB-468 TNBC cells. Biodistribution studies in Balb/c mice bearing MDA-MB-468 xenografts demonstrated specific uptake of both ^125^I- and ^99m^Tc-labeled Ec1 in TNBC tumors. ^125^I-labeled Ec1 had appreciably lower uptake in normal organs compared with ^99m^Tc-labeled Ec1, which resulted in significantly (*p* < 0.05) higher tumor-to-organ ratios. The biodistribution data were confirmed by micro-Single-Photon Emission Computed Tomography/Computed Tomography (microSPECT/CT) imaging.

A new minigastrin (MG) analog (DOTA-DGlu-Pro-Tyr-Gly-Trp-(*N*-Me)Nle-Asp-1Nal-NH_2_ with site-specific amino acid substitutions and stabilized against enzymatic degradation) and possible metabolites were synthesized and investigated in preclinical studies by Hormann et al. A biodistribution study of the radiolabeled peptide in BALB/c mice showed low background activity, preferential renal excretion and prolonged uptake in CCK2R-expressing tissues. The in vivo stability study of the radiolabeled peptide was >56% intact radiopeptide in the blood of BALB/c mice 1 h post-injection. High CCK2R affinity and cell uptake were confirmed only for the intact peptide, whereas enzymatic cleavage within the receptor-specific C-terminal amino acid sequence resulted in a complete loss of affinity and cell uptake.

[^68^Ga]Ga-DOTA-AmBz-MVK(Ac)-OH and its derivative, [^68^Ga]Ga-DOTA-AmBz-MVK(HTK01166)-OH, coupled with the PSMA-targeting motif were synthesized and evaluated by Bendre et al. to determine if they could be recognized and cleaved by the renal brush border enzymes. [^68^Ga]Ga-DOTA-AmBz-MVK(Ac)-OH was effectively cleaved specifically by neutral endopeptidase (NEP) of renal brush border enzymes at the Met-Val amide bond, and the radio-metabolite [^68^Ga]Ga-DOTA-AmBz-Met-OH was rapidly excreted via the renal pathway with minimal kidney retention. [^68^Ga]Ga-DOTA-AmBz-MVK(HTK01166)-OH retained its PSMA-targeting capability and was also cleaved by NEP. It appears that MVK can be a promising cleavable linker for use to reduce kidney uptake of radiolabeled DOTA-conjugated peptides and peptidomimetics.

Halik and coworkers developed and evaluated two novel ^68^Ga and ^177^Lu-labeled chelate conjugates for their lipophilicity and stability in human serum. Additionally, the fully stable conjugates were examined in molecular modeling with a human neurokinin 1 receptor structure and in a competitive radioligand binding assay using rat brain homogenates. This initial research is in the conceptual stage to give potential theranostic-like radiopharmaceutical pairs for the imaging and therapy of neurokinin 1 receptor-overexpressing cancers. 

Lin et al. evaluated the therapeutic efficacy of ^188^Re-liposome on Hypopharyngeal Cancer (HPC) tumors using bioluminescent imaging followed by next-generation sequencing (NGS) analysis to address the deregulated microRNAs and associated signaling pathways. Repeated doses of ^188^Re-liposome were administered to tumor-bearing mice, and the tumor growth was suppressed after treatment. It was concluded that the ^88^Re-liposome could suppress the HPC tumors in vivo, and the therapeutic efficacy was associated with the deregulation of microRNAs that could be considered as a prognostic factor.

Four review articles are included in this Special Issue. The first review by Kumar and Ghosh provides a comprehensive review of the physical properties of iodine and iodine radionuclides, production processes of ^124^I, various ^124^I-labeling methodologies for molecules and biomolecules, peptides, proteins, protein fragments, and mAbs, and the development of ^124^I-labeled immunoPET imaging pharmaceuticals for various cancer targets in preclinical and clinical environments. The second review by Alluri et al. provides an overview of the development of positron emission tomography (PET) radiotracers for in vivo imaging of the adrenergic receptors (ARs) system in the brain. The third article by Eychenne et al. focuses on the development of radiolabeled somatostatin analogs (SSAs) to visualize the distribution of receptor overexpression in tumors and radiotherapy of many solid tumors, especially gastro-entero-pancreatic neuroendocrine tumors (GEP-NET). The fourth review by Gomes et al. focuses on the use of pyrazoles as suitable scaffolds for the development of ^18^F-labeled radiotracers for PET imaging in the last 20 years.

In summary, radiolabeled compounds play an important role in the diagnosis and treatment of various cancers. Tremendous progress has been made in discovering, developing, and registering with the FDA numerous radiopharmaceuticals for imaging and therapy by targeting various receptors in cancer. The impact of radiolabeled compounds in academia and industry is profound, and this continuous research tends to develop more novel compounds for unmet needs. Contributions from this Special Issue in Molecules will add significantly to the field of Radiopharmaceuticals.

Finally, I would like to thank the authors for their contributions to this Special Issue, the reviewers for their critical review in evaluating the submitted articles, and the editorial staff of Molecules, especially the Assistant Editor of the journal, Emity Wang, for her kind assistance during the preparation and release of this Special Issue.
